# A Dual Mode Propulsion System for Small Satellite Applications^‡^

**DOI:** 10.3390/aerospace5020052

**Published:** 2018-05-02

**Authors:** Kevin R. Gagne, M. Ryan McDevitt, Darren L. Hitt

**Affiliations:** 1 Department of Mechanical Engineering, University of Vermont, Burlington, VT 05405, USA; kgagne@blueorigin.com; 2 Benchmark Space Systems Inc., South Burlington, VT 05403, USA; rmcdevitt@benchmark-space.com

**Keywords:** micropropulsion, microthruster, small satellites, additive manufacturing, hydrogen peroxide, monopropellant

## Abstract

This study focused on the development of a chemical micropropulsion system suitable for primary propulsion and/or attitude control for a nanosatellite. Due to the limitations and expense of current micropropulsion technologies, few nanosatellites with propulsion have been launched to date; however, the availability of such a propulsion system would allow for new nanosatellite mission concepts, such as deep space exploration, maneuvering in low gravity environments and formation flying. This work describes the design of “dual mode” monopropellant/bipropellant microthruster prototype that employs a novel homogeneous catalysis scheme. Results from prototype testing are reported that validate the concept. The micropropulsion system is designed to be fabricated using a combination of additively-manufactured and commercial off the shelf (COTS) parts along with non-toxic fuels, thus making it a low-cost and environmentally-friendly option for future nanosatellite missions.

## 1. Introduction

Next generation small satellites, also known as nanosats, have masses significantly lower than standard satellites, often in the range of 1–10 kg. These satellites are being developed for numerous applications related to research, defense and industry. An important subcategory of nanosats are those built on the CubeSat specification, which allows for cube-shaped satellites, as small as 10 cm per side, with masses as low as 1.33 kg [1,2]. CubeSats offer a low cost alternative to traditional satellites due to their low Size, Weight and Power (SWaP). Traditionally, CubeSats have had few options for on-board propulsion, but recent advances in fields such as additive manufacturing, microfluidics, MicroElectroMechanical Systems (MEMS), low power microelectronics, high efficiency solar cells, and advanced materials, as well as an increased interest from the commercial sector, are leading to new propulsion developments. Future CubeSats, incorporating these advances, could enable scientific missions that would be impossible or financially prohibitive using traditional satellites.

There are many potential advantages of incorporating propulsion systems on future CubeSat missions. Some of these advantages include extended mission lifetime, maneuvering ability, formation flying, proximity operations, fine attitude control, drag make-up, and de-orbiting [3]. Today, NASA’s CubeSat Launch Initiative (CSLI) plans on providing low cost access to space for research purposes for over 100 CubeSats developed by both the United States government as well as non-profit organizations. Without on-board propulsion systems, these missions are expected to have a lifespan of approximately 90 days before they fall to Earth and burn up in the atmosphere [4]. Through the integration of propulsion systems, lifetimes on missions similar to those of the CSLI missions could be greatly extended providing the government and independent researchers the ability to collect significantly more data.

There are numerous types of CubeSat propulsion systems in active development, including those based on solar sails, electrodynamic tethers, ion electrospray, miniaturized Hall thrusters, solid rocket motors, liquid chemical thrusters, cold gas thrusters, and pulsed plasma thrusters. Recent reviews of propulsion technologies and trends for small satellites and cubesats can be found in Refs. [5–9]. While many of these technologies offer great promise, the high cost of manufacturing these systems along with the need for advancements in electric power generation and storage, thermal management, and power processing unit (PPU) technologies currently hinder the implementation of these propulsion systems on CubeSats. A reliable, low-cost, easily manufactured propulsion system is therefore necessary for CubeSat propulsion to be widely implemented.

Among the most appealing of these technologies are chemical propulsion systems, owing to their combination of simplicity, reliability, and low power requirements. Liquid propulsion systems are of particular interest both as primary propulsion and attitude control thrusters, as demonstrated by recent efforts such as the University of Vermont Discrete Monopropellant Microthruster [10] and the ESA-sponsored PRECISE project [11], which both use the catalyzed decomposition of a monopropellant to generate thrust. Each of these thruster initiatives, however, has reported challenges associated with the performance of the catalytic chamber due to scaling effects.

In a previous study by the authors [12], a method for decomposing a monopropellant without a catalytic chamber was proposed that could address these challenges. Using this method, the monopropellant and an aqueous catalyst are fed into a mixing chamber to initiate decomposition, with the products ejected through a converging–diverging nozzle. A significant benefit of the homogeneous catalysis process—and the key contribution of this work—is the ability to operate in two distinct modes, a “pseudo-monopropellant” mode and a “bipropellant” mode. These different modes of operation are achieved by varying the the flow rates of the catalyst solution and hydrogen peroxide. By changing the ratio of catalyst solution to hydrogen peroxide in the mixing and reaction chamber, the resulting thrust and impulse bit can be altered and optimized for specific situations. The dual mode propulsion system offers simultaneous capabilities for both attitude control and primary propulsion for CubeSat-class satellites. Attitude control operations could include station keeping, detumble, and reaction wheel desaturation, whereas primary propulsion allows for CubeSat orbital maneuvering. Aside from the operational flexibility, a second major advantage of the dual mode system is the ability to use the same propellants, piping, and valving for both high and low thrust applications. It is noteworthy that the dual-mode propulsion concept has already been realized on the larger (or “macro”) scales with hypergolic propulsion systems. In contrast, however, implementing this design on the miniaturized scale associated with small satellites is inherently problematic due to the lack of flow mixing at the low Reynolds numbers and the emergence of capillary and surface wetting effects. In this sense, our implementation represents a new development in spacecraft propulsion.

One potential application for a dual-mode miniaturized propulsion system is that of cubesat-based exploration of near-Earth objects, asteroids and comets [13–16]. As these small celestial bodies typically have weak and irregular gravitational fields, they present an interesting propulsion challenge. Attitude control and station keeping for a cubesat orbiting an asteroid, say, will require low thrust levels and short duration firings that are better suited for a monopropellant thruster. To transit from Earth to the asteroid, however, a high-thrust, high-specific-impulse bipropellant thruster would be desirable, to reduce the wet mass of the cubesat at launch. A dual-mode propulsion system allows the satellite to operate in the most efficient mode for the mission profile without the need for multiple independent propulsion systems.

In this work, the design overview of a dual-mode propulsion system is presented along with supporting results from preliminary experiments demonstrating the concept. This article describes the selection of the fuel/catalyst combination (in both monopropellant and bipropellant operation), development of the monopropellant attitude control thrusters, and development of the dual-mode primary divert thrusters.

## 2. Propulsion Mechanism Overview

For a propulsion system based on a hydrogen peroxide monopropellant, the operation is based on the idealized chemical decomposition reaction that proceeds according to the reaction described in Equation (1).

(1) $$H_{2}O_{2}(l)+nH_{2}O(l)\to \;\frac{1}{2}O_{2}(g)+(n+1)H_{2}O(g)+\text{heat}$$

**Figure 1.**
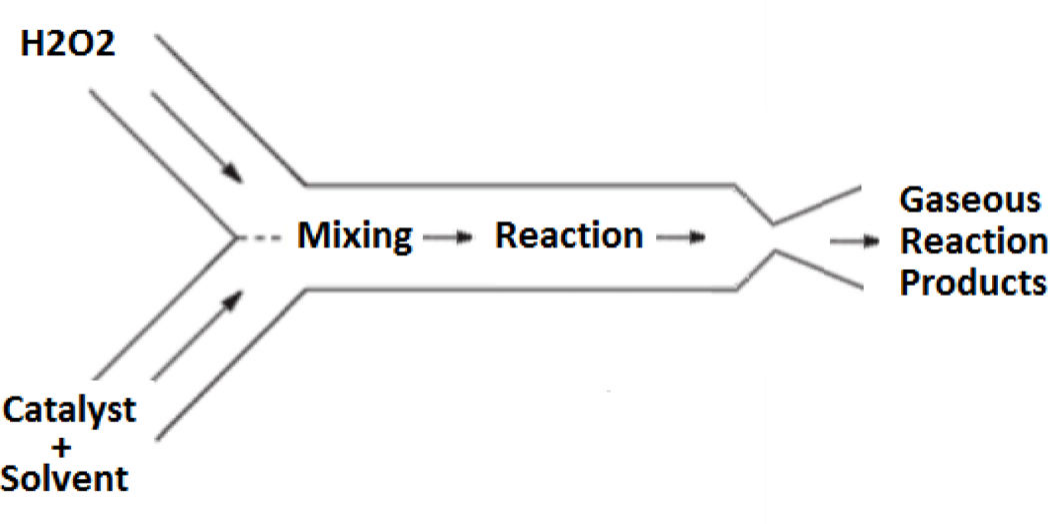
This drawing shows the general principle of homogeneous catalysis and how it is applied in the system outlined in this paper. High test hydrogen peroxide and catalyst are both fed into a mixing chamber from their respective storage vessels. In the mixing and reaction chamber, the chemical reaction takes place, producing gaseous reaction products. The gases are then expelled through a converging–diverging nozzle.

Here, *n* represents a mole fraction of water present in the hydrogen peroxide fuel; typically, the hydrogen peroxide concentrations ranges 85–90% by mass. In this propulsion design, the decomposition is achieved using a homogeneous catalysis approach wherein the catalyst is dissolved in a liquid solvent and reacts with the monopropellant in the liquid phase. The resulting exothermic reaction produces high temperature gaseous products that are subsequently passed through a converging–diverging supersonic nozzle to produce thrust. A high-level representation of this process is shown schematically in Figure 1.

The detailed operation of this propulsion concept can be broken down into three stages: storage, mixing and reaction, and expulsion. First, in the storage stage, the catalyst solution and hydrogen peroxide are both stored separately in chemically compatible vessels. In this system, the storage vessels are pressurized by the slow, controlled *auto-decomposition* of hydrogen peroxide, resulting in a pressure increase in the storage vessels without the need for an external pressure source. Not only does this approach mitigate the need for a separate pressurization system aboard the cubesat, but it also satisfies current NASA regulations for pressurized elements on cubesats during launch.

The second stage, mixing and reaction, occurs when the hydrogen peroxide and catalyst solutions are allowed to flow into the mixing and reaction chamber of the thruster. Here, the catalyst solution is mixed with the hydrogen peroxide resulting in a fast decomposition reaction. This reaction produces steam, oxygen, and heat. The heat released by the decomposition reaction vaporizes the catalyst solvent. Due to the high temperature created by the decomposition reaction, the catalyst solvent vapors react with the oxygen resulting in a *secondary combustion reaction*. The secondary combustion reaction releases additional thermal energy into the flow thereby increasing the *I*_*sp*_ of the propulsion system; *this aspect represents a significant and novel aspect of this design*.

After the reactions have occurred in the mixing and reaction stage, the hot product gases enter the third stage of the propulsion system, the converging–diverging nozzle. The decrease in mass density of the gaseous reaction products compared to the density of the liquid reactants results in substantial pressure increase in the mixing and reaction chamber. This pressure drives the reaction products through the converging–diverging nozzle to achieve supersonic conditions and thereby generate the thrust for the device.

At the lowest operational ratio of catalyst solution to hydrogen peroxide, the thrust produced will approach the results of a monopropellant thruster; this is the “pseudo-monopropellant” mode of operation. When operating in pseudo-monopropellant mode, the energy released approaches that of a monopropellant hydrogen peroxide system. In this mode, approximately 586 calories of heat per gram of 85% hydrogen peroxide is released and the adiabatic flame temperature during operation is 886 K [17].

In contrast, by increasing the ratio of catalyst solution to hydrogen peroxide to a stoichiometric ratio allows all of the oxygen produced by the hydrogen peroxide decomposition to participate in a subsequent combustion reaction with the catalyst solvent. Coupling the combustion of the catalyst solvent with the decomposition of hydrogen peroxide results in a dramatic increase in thrust. This mode of operation is similar to a traditional bipropellant system, and is thus referred to as the “bipropellant mode” of operation. When the decomposition of hydrogen peroxide is coupled with the combustion of a catalyst solvent, the overall reaction products are carbon dioxide and steam and the total number of gas particles created by the reaction is increased. The addition of a stoichiometric ratio of energy-dense solvent significantly increases the energy evolved resulting in an increased adiabatic flame temperature. In comparison to the pseudo-monopropellant mode, the use of a stoichiometric mixture of hexanol and pure hydrogen peroxide results in an energy density of approximately 1050 calories per gram of mixture with an adiabatic flame temperature of 2670 K.

The theoretical differences between the pseudo-monopropellant mode and bipropellant mode are summarized in Table 1.

## 3. Propellant and Catalyst/Solvent Selection

The CubeSat Design Specification, which creates a standard that ensures that CubeSats and their launch vehicle are compatible, sets strict limiting factors for the characteristics of CubeSat propulsion systems. Along with overall mass limitations for a 1U and 3U CubeSat at 1.33 kg and 4 kg, respectively, the maximum pressure contained in any component is limited to 1.2 atmospheres. Due to the high energy density, low vapor pressure, and nontoxic characteristics of hydrogen peroxide, it is a promising choice for use in a CubeSat propulsion system. In comparison to many other compounds used in propulsion today, such as hydrazine and oxides of nitrogen, high test hydrogen peroxide (HTP) has low toxicity and is considered a “green” monopropellant. The volume specific impulse of 90% HTP is greater than most comparable green propellants currently available. Another advantage of using HTP as a propellant is the benign nature of the products of hydrogen peroxide decomposition, steam and diatomic oxygen; unlike other promising high-energy green monopropellants such as ADN, HAN, and HNF, the decomposition of HTP results in products with relatively low molecular weight. This is an advantage because the operational temperature of the exhaust gases in HTP thrusters can be maintained much lower than that of other “green” propellants resulting in decreased materials and manufacturing costs [18]. Due to these characteristics, HTP is a relevant propellant with a significantly reduced cost of manufacturing, storage, and handling when compared to many other fuels on the market today.

To decompose the HTP, a catalyst is used to accelerate the rate of the decomposition reaction. Previous work using heterogeneous ruthenium-oxide (RuO_2_) nanostructures as an in situ microchannel catalyst showed signs of incomplete decomposition, most likely caused by the inability of the HTP to wet the surface of the catalyst [10,19]. For the present work, homogeneous catalysis is used. This approach has many advantages over heterogeneous catalysis. Perhaps the most compelling advantage is the ability to use additive manufacturing for the device fabrication, decreasing the manufacturing cost and lead time. Two aqueous catalysts were chosen based on the available literature available: ferric chloride (*Fe*(*III*)*Cl*_3_) and ferrous chloride (*Fe*(*II*)*Cl*_2_) [20]. Due to the ionic nature found in the bonds of both the catalysts, they are readily disassociated into their constituent ions; this results in a high concentration of iron ions in the solution that catalyzes the decomposition of hydrogen peroxide [21].

An advantage of using an ionic compound as the catalyst for the reaction is the ability to use a polar solvent in the catalyst solution. Many polar solvents have high energy densities, low vapor pressures, and include many low toxicity compounds. Among such solvent options are water and alcohols. Several different catalyst solvents were tested in this study, each with a varying degree of polarity. The catalyst solvents were chosen based on several criteria: the ability to dissolve the catalyst, the miscibility with hydrogen peroxide, energy density, auto ignition temperature, and vapor pressure. Based on published data or estimates of these characteristics, six solvents of interest were chosen: hexanol, 2-propanol, ethanol, methanol, hexane, and water. Water was chosen as a baseline, non-reactive solvent. The polarity of the catalyst solvent allows the catalyst solution to be miscible with hydrogen peroxide. Although hexane’s dipole moment is significantly lower than the other solvents of interest, it was chosen to determine the degree of polarity necessary to dissolve the catalyst solutes. Nonpolar catalyst solvents tend to be immiscible with hydrogen peroxide and would greatly hinder mixing and hence unacceptably slow reactions in the thruster mixing chamber.

The fast rate of decomposition of the hydrogen peroxide allows for exhaust gases to be generated on demand while maintaining high flow rates during steady state operation. Therefore, it is important that the catalyst used for the reaction results in sufficiently fast reaction rates. The use of homogeneous catalysis eliminates catalyst beds completely, resulting in the elimination of problems caused by a contaminated catalyst surfaces (i.e., fouling). The constant mixing of fresh catalyst in the homogeneous catalysis design means that catalyst degradation over time is not an issue of concern in this design.

A key advantage of using the fuel as the catalyst solvent is the ability to vary the reaction kinetics by varying the ratio of hydrogen peroxide to fuel. If the system is run at the full stoichiometric ratio (1:9 fuel-to-oxidizer ratio for 2-propanol), the system operates as a bipropellant thruster with complete combustion of the fuel. By reducing the fuel injected into the reaction chamber, the system will transition to pseduo-monopropellant mode, where the majority of the reaction energy is coming from the exothermic decomposition of the hydrogen peroxide. The result of this control is the ability to use the same thruster, and related hardware, with a number of different operating profiles.

When the decomposition of hydrogen peroxide is coupled with the combustion of a catalyst solvent, the overall reaction products are carbon dioxide and steam. The total number of gas particles created through the combustion reaction is increased. This, along with an increase in the enthalpy of reaction results in larger stagnation pressures and temperatures upstream of the nozzle. The bipropellant mode of the microthruster design takes advantage of these properties resulting in an increased thrust and impulse bit.

When the desired values of thrust and impulse bit are below the minimum threshold for bipropellant operation, the ratio of catalyst solution to hydrogen peroxide is decreased significantly. This causes the enthalpy of reaction and product stream compositions to shift towards that of a hydrogen peroxide monopropellant thruster. The overall performance shift can be used when small adjustments are necessary, such as attitude control and small delta-V maneuvers.

For operation in a deep space environment, it is worth noting the freezing point of 90% propellant grade hydrogen peroxide is *−*10 °C. Due to the freezing point depression caused by the colligative properties in the catalyst solution, the freezing points are depressed far below the freezing point of hydrogen peroxide for each combination of catalyst and solvent of interest. As a result, no additional heat maintenance is required for the described bipropellant system than a hydrogen peroxide monopropellant thruster.

The decomposition of hydrogen peroxide is extremely exothermic; when used in monopropellant mode, the energy released is approximated by the energy released through only the decomposition of hydrogen peroxide, 586 calories of heat per gram of 85% HTP decomposed with a resulting adiabatic flame temperature of 886 K [17]. The addition of an energy-dense solvent for the catalyst solution allows a co-combustion reaction to occur, increasing the energy released by the reaction, resulting in an increased adiabatic flame temperature. In comparison to the monopropellant mode, the use of a stoichiometric mixture of hexanol and pure hydrogen peroxide results in an energy density of approximately 1050 calories per gram of mixture with an adiabatic flame temperature of 2670 K. During bipropellant mode of operation, much more chemical energy will be liberated resulting in increased thrust compared to monopropellant mode.

**Figure 2.**
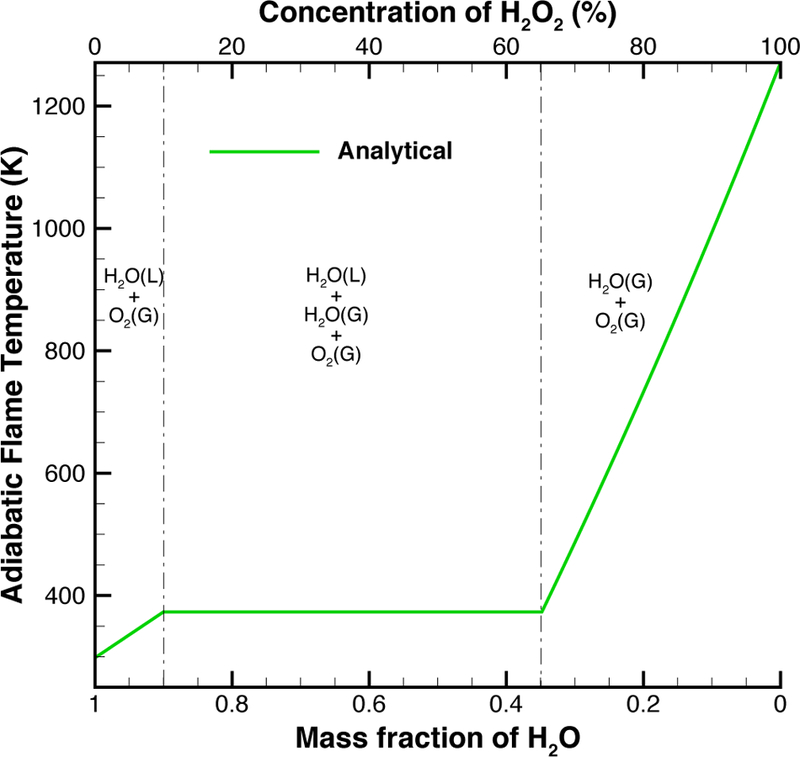
Plot of the adiabatic flame temperature of hydrogen peroxide as the concentration is varied. To reach complete decomposition, the concentration of hydrogen peroxide in the monopropellant/aqueous catalyst solution must exceed 65%.

The addition of water to the hydrogen peroxide through the use of aqueous ferric chloride solution decreases the adiabatic flame temperature, as shown in Figure 2, but increases the total mass flow rate through the nozzle. The decrease in temperature can be negated by increasing the purity of hydrogen peroxide. By decreasing the water in the fuel, the same adiabatic flame temperature can be achieved using the aqueous catalyst solution. For example, the use of 98% hydrogen peroxide as fuel would allow a mixing ratio of approximately 15% catalyst solution before the adiabatic flame temperature dipped below 886 K.

**Figure 3.**
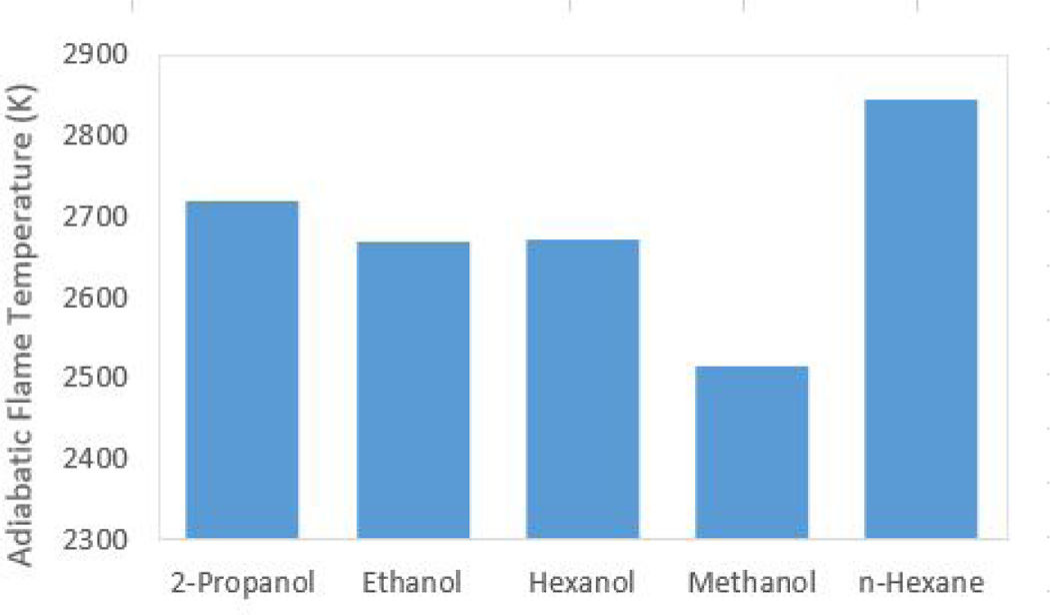
Adiabatic flame temperatures of a stoichiometric mixture of hydrogen peroxide and various catalyst solvents.

By replacing water solvent with a fuel at a stoichiometric ratio of fuel to hydrogen peroxide, the adiabatic flame temperature of the mixture is increased significantly. If ethanol, methanol, 2-propanol, hexane, or hexanol are used as the catalytic solvent, the resulting adiabatic flame temperatures are significantly higher as shown in Figure 3. The adiabatic flame temperature increase when compared to using water as a solvent is a direct consequence of the substantial increase of chemical energy released through the combustion of the fuel in combination with the decomposition of hydrogen peroxide.

### 3.1. Monopropellant/Catalyst Solution Suitability Results

Results from monopropellant and bipropellant data were divided into three categories based on the observed reaction delay time. The “long delay” category contained solutions with an observed delay of greater than one second, the “short delay” category contained solutions with a delay less than or equal to one second, and the “no delay” category contained solutions with no observed delay before the reaction. The reactions were classified into “fast” or “slow” reactions if the reaction after the delay period was shorter or longer than 0.5 s. Using data from the reaction observations, each solvent was determined to be either fit or unfit for use in this application. If the solution had no delay and a fast reaction, it was put into the fit category; if the solution had a delay longer than 1 s or a reaction longer than 0.5 s after the delay, the solution was unfit. These data are shown in combination with collected solubility data in Table 2.

### 3.2. Catalyst Solution Selection

The viable catalyst solutions were chosen by combining the solubility data and the chemical reactivity data. Viable catalyst solutions, which possess characteristics necessary for use in a functioning thruster, were chosen as solutions that were homogeneous and noncolloidal at −10 °C, and have no observed delay followed by a fast decomposition reaction during reactivity testing. Through this process, the original 36 possible catalyst solutions were further reduced to seven viable catalyst solutions.

**Figure 4.**
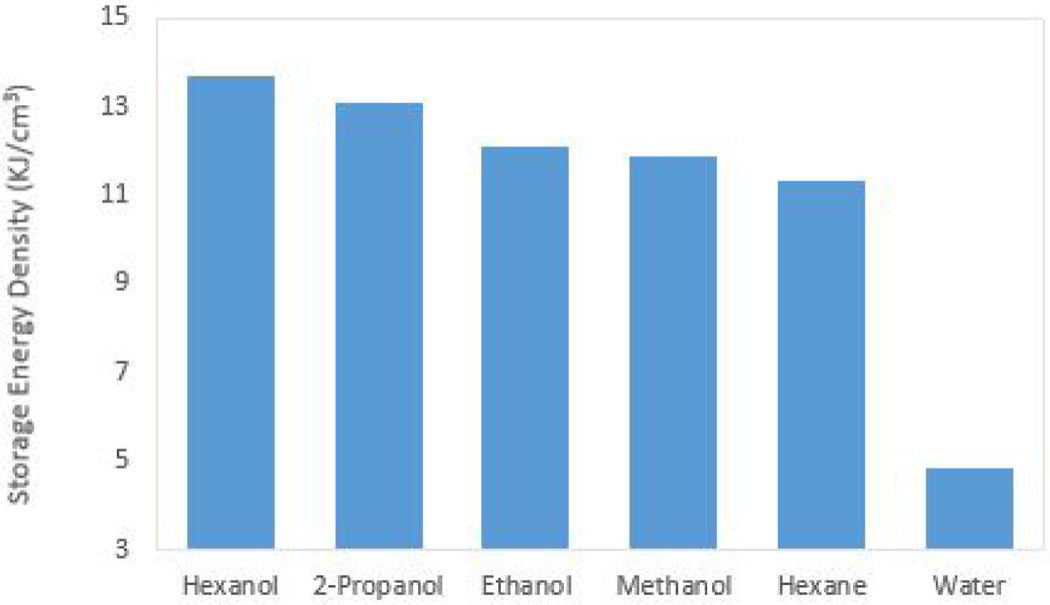
The energy density of the stoichiometric ratio of hydrogen peroxide and solvent. Higher energy densities allow for smaller total storage volume leading to decreased system footprint.

Other characteristics that impact the catalyst solution effectiveness are the energy density of the chemical storage space, the solute concentration, and the relative theoretical maximum specific impulse. Each of these characteristics contribute to the thruster’s ability to provide consistent and reliable performance. The energy density of interest is the total energy per unit volume of the stoichiometric ratio of catalyst solution to hydrogen peroxide. When operating in bipropellant mode, the volumetric ratio of hydrogen peroxide and solvent differs between solvents due to the combustion chemistry of the reaction. When the volumetric ratios of hydrogen peroxide and solvent are taken into consideration, the solvent with the highest combined energy density will provide the thrust system with the highest delta-V per unit of combined hydrogen peroxide and catalyst storage space. This is important because of the tight volume constraints imposed by the CubeSat platform. Figure 4 shows the energy density of each solvent.

There is a possibility that the catalyst will recrystallize and form a precipitate layer of catalyst on the walls of the nozzle during thruster operation. Due to this, the concentration of the catalyst in the solvent has the possibility to affect the performance of the thruster over its operational lifetime. This problem is mitigated by using the minimum catalyst concentration in the catalyst solution while maintaining high reaction kinetics in the device. Minimizing the mass of catalyst also decreases the total mass of the system.

**Figure 5.**
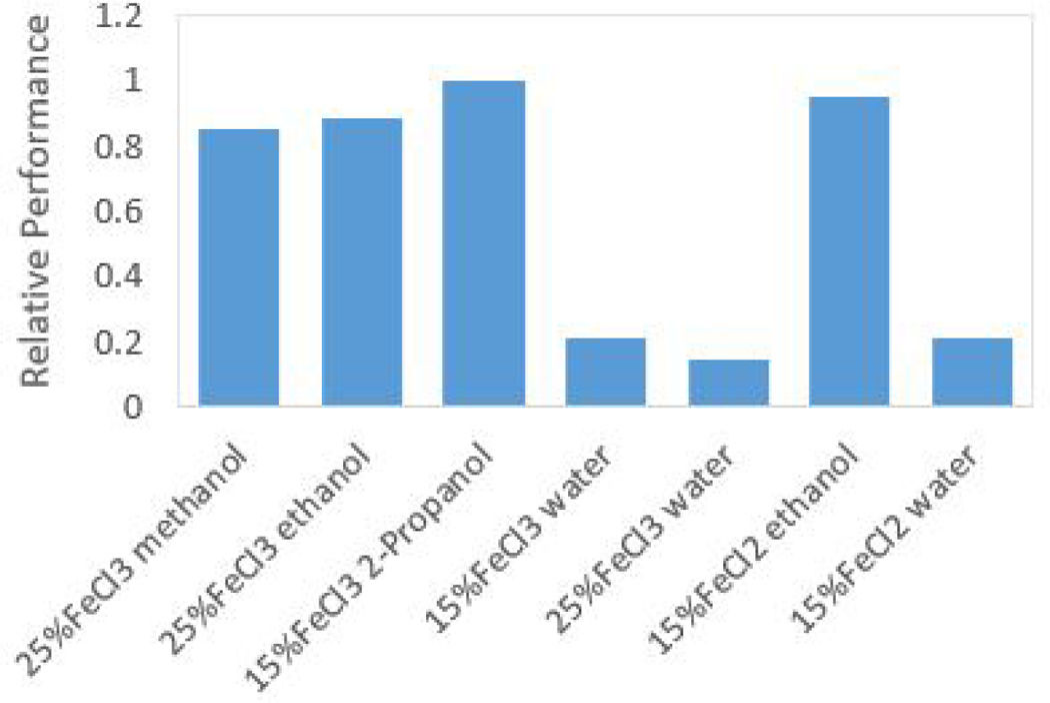
The relative expected performance of each solvent of interest based on three major performance and reliability characteristics including storage energy density, relative specific impulse, and solute concentration.

To characterize the expected performance of each of the catalyst solutions, the theoretical relative specific impulse of each was calculated. Theoretical relative specific impulse is the theoretical maximum achievable specific impulse for each solvent normalized by the maximum achievable specific impulse of 85% hydrogen peroxide and water. By determining which solvents have the highest relative specific impulse, the performance of the solvents can be directly compared. Figure 5, which is a plot of expected bipropellant performance, shows the rankings of relative expected performance of each catalyst solution based on the relative specific impulse (*I*_*SPR*_), relative chemical storage energy density (*U*_*R*_), and relative solute concentration (*Conc*_*R*_). The expected performance was characterized using an empirical, weighted formula given by :

(2) $$\text{Expected Performance}=\text{5}U_{R}\;+\text{5}I_{SPR}\;-1.\text{5}Conc_{R}$$

The relative expected performance was then determined by normalizing the expected performance with the highest performing solvent, in this case, 2-propanol.

Based on this metric, the catalyst solution predicted to provide the thruster system with the highest performance is 15% ferric chloride in 2-propanol. This solution was chosen by taking relative specific impulse, average storage energy density, and catalyst concentration into consideration. This solution showed promising results in preliminary chemical reaction and solubility testing. It was determined that this solution will take advantage of high performance of the combustion of 2-propanol with a relatively low concentration of catalyst, increasing thrust and reliability.

## 4. System Design Overview

**Figure 6.**
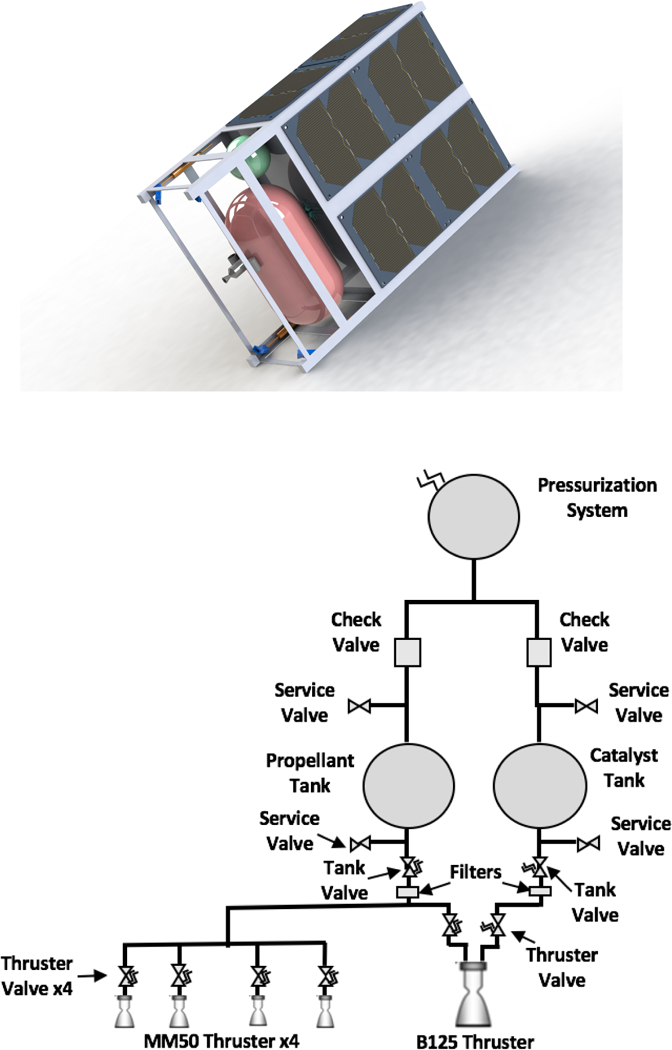
Summary of the B125 Propulsion System in development: (**top**) a solid model rendering of an envisioned 6U cubesat architecture; and (**bottom**) a system schematic diagram. Note that the tank configuration depicted above is not the tank configuration for the laboratory testing described in this study.

**Figure 7.**
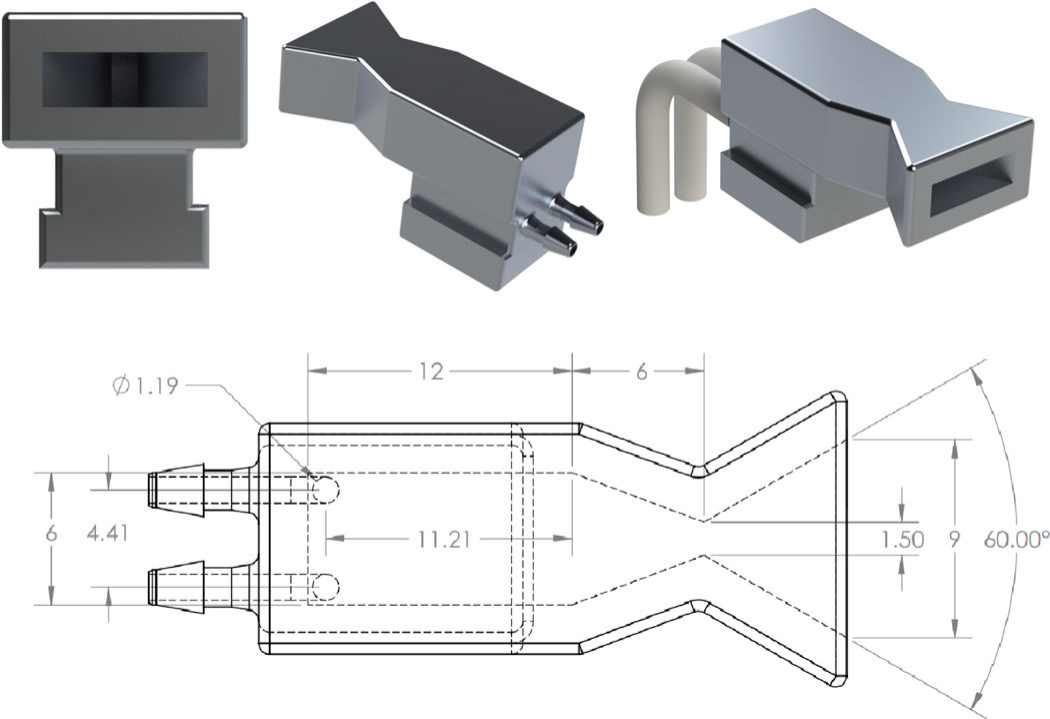
Schematic of the 50 mN attitude control thruster. Dimensions are in mm.

**Figure 8.**
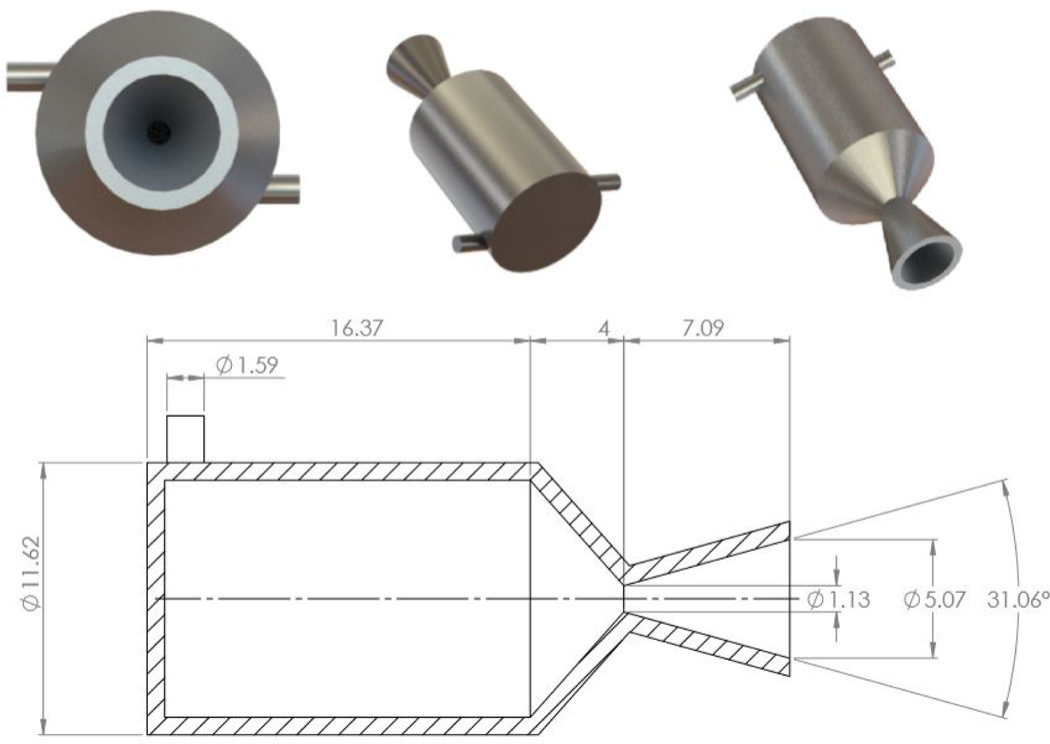
Schematic of the 1.25 N primary driver thruster. Dimensions are in mm.

With the solvent/oxidizer and fuel identified, a proposed system was developed to offer primary propulsion and attitude control for a 6U CubeSat. The envisioned system, illustrated in Figure 6, uses one 1.25 N bipropellant thruster for primary propulsion and four 50 mN monopropellant thrusters for attitude control. A summary of the key design parameters for the two different propulsion systems appears in Table 3. The primary “driver thruster” is mounted on the spacecraft centerline with the thrust axis pointed through the nominal spacecraft center of mass. The four attitude control thrusters are canted at the corners of the spacecraft frame to maximize the moment arm to the spacecraft center of mass, and thus the control authority. The design of the attitude control thruster is depicted in Figure 7 and that of the driver thruster appears in Figure 8. The remaining propulsion system components consist of the oxidizer (H_2_O_2_) and fuel (2-propanol) tanks, the valves and manifold. Specific details of the two different thrusters are provided in the following subsections.

### 4.1. Attitude Control Thrusters

The attitude control thrusters are additively manufactured out of stainless steel using Direct Metal Laser Sintered (DMLS). This manufacturing process allows the plenum, reaction chamber, and converging–diverging nozzle to be printed in a single step, reducing production costs and simplifying production. Decomposition of *H*_2_*O*_2_ requires no preheating, and the decomposition temperature is significantly below the melting point of stainless steel, so no thermal management is required during continuous operation of the thrusters. Each attitude control thruster is paired with two miniature solenoid valves, which control the HTP and fuel-catalyst injection. Upon entering the reaction chamber, the two fluids mix and the HTP spontaneously decomposes. By maintaining the mixture ratio significantly below the stoichiometric level, these thrusters operate as a monopropellant thruster. The decomposition gases are ejected through a linear converging–diverging micronozzle, with a 30° half-angle. This configuration was selected as it provides a balance between minimizing the volume the nozzles occupy and the expected performance of the miniaturized supersonic nozzle including viscous effects at lower Reynolds numbers [22].

### 4.2. Primary Driver Thruster

The primary thruster is additively manufactured out of Inconel using DMLS. Inconel, a nickel-chromium alloy, is selected based on its corrosion resistance and ability to operate in extreme environments of pressure and heat. In the present case, the operating temperature of the primary thruster is significantly above the melting point of stainless steel. Like the attitude control thrusters, the use of DMLS allows the inlet plenum, reaction chamber, and converging–diverging nozzle to be printed in a single piece. Here, owing to the larger Reynolds number of the flow, a conventional conical nozzle with a 15° half-angle is used.

The primary thruster uses two miniature solenoid valves to control the fuel and oxidizer. By pulsing the valves, the flow rates can be varied to operate either in bipropellant or pseudo-monopropellant mode. The system has no active thermal management, relying on radiative cooling, which limits continuous firing of the thruster in bipropellant mode to short bursts of under 30 s followed by a 5 min cooling period. In pseudo-monopropellant operation, the system can operate continuously with no thermal concerns.

## 5. Experimental Device Testing and Characterization

Due to the significant difference in thrust levels between the attitude control thrusters and the primary driver thruster, different measurement techniques were required for performance characterization. Three indirect measurement techniques were used to demonstrate the flow characteristics of the 50 mN attitude control thrusters: Schlieren imaging, thermal imaging, and exit plane temperature analysis. The thrust of the 1.25 N primary driver thruster was measured directly on a thrust stand. Specific descriptions of the methods used follow.

### 5.1. Schlieren Imaging of Micronozzle Operation

To assess the micronozzle performance, Schlieren photography was performed using ambient temperature compressed air. The house air stagnation pressure was measured to be approximately 55 psig. According to the compressible flow relation the ratio of the stagnation pressure to outlet pressure necessary to achieve sonic flow at the throat was 1.57 (using γ = 1.4 for air), resulting in a necessary minimum stagnation pressure of approximately 38 psig when vented directly to the laboratory. Due to this, the pressure drop over the nozzle resulting from the supplied house air was determined to be theoretically large enough for supersonic flow in the divergent section of the nozzle.

**Figure 9.**
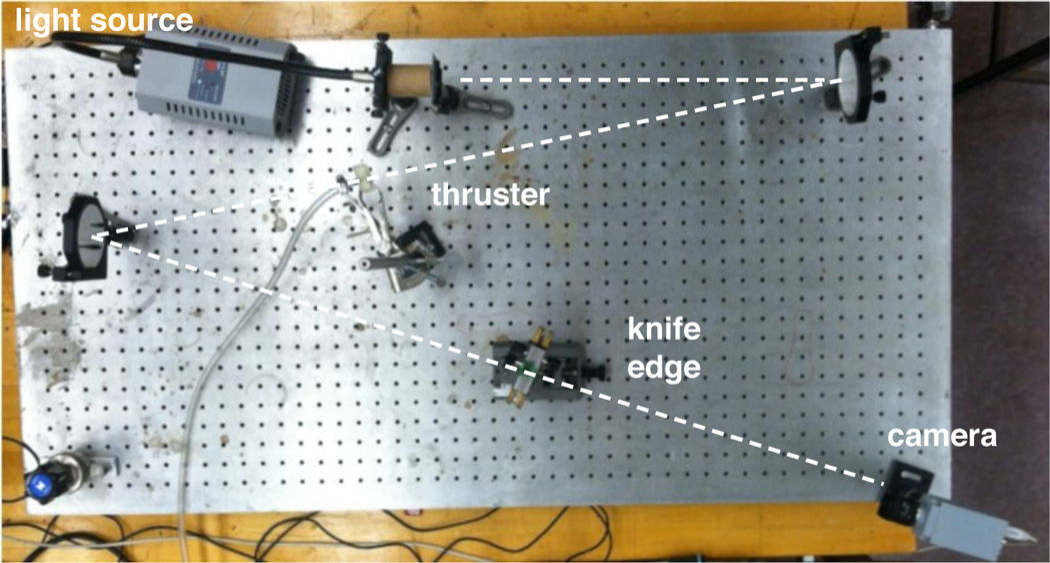
Optical table with a “z-type” Schlieren photography system used for flow visualization of the nozzle plume. The light source is in the top left and the camera is in the bottom right; the light path is the dashed line. The focal length of the mirror is 1 m.

The Schlieren photography apparatus is shown in Figure 9. The Schlieren apparatus used for this experiment was a Z-type system consisting of a bright light source that passes through a 0.25 mm hole, directed at a mirror that is placed one focal length away from the light source. The first mirror collimates the light and redirects the collimated light over the test region, where the nozzle was placed. After passing through the test region, another mirror refocuses the light towards the knife’s edge, in this case the edge of a razor blade, which is located one focal length from the second mirror. A camera located behind the knife edge was used to capture the Schlieren photographs.

**Figure 10.**
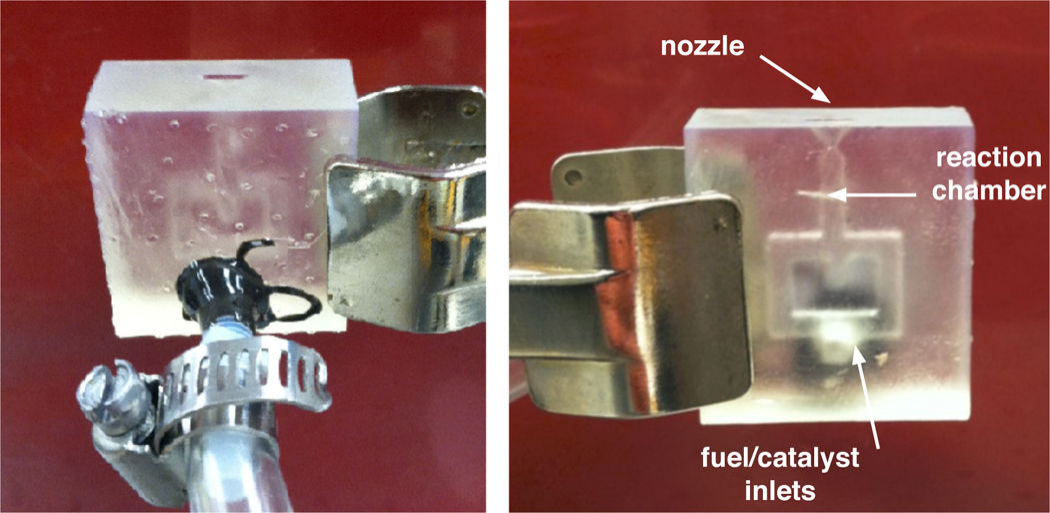
Nozzle with 30 degree half angle used for verification of supersonic flow in the test region of the in-house Schlieren photography system. The photo on the left shows the house air connection which was secured with epoxy, the photograph on the right shows the simplified flow network and nozzle half angle.

For testing, a simplified flow network design was created that eliminated one of the inlets from the nozzle design, leaving one inlet for the house air line. A photograph of the nozzle that was used in the Schlieren imaging is shown in Figure 10. The nozzle was additively manufactured using Formlabs Clear Photopolymer Resin, formulation FLGPCL02.

### 5.2. Thermal Imaging

**Figure 11.**
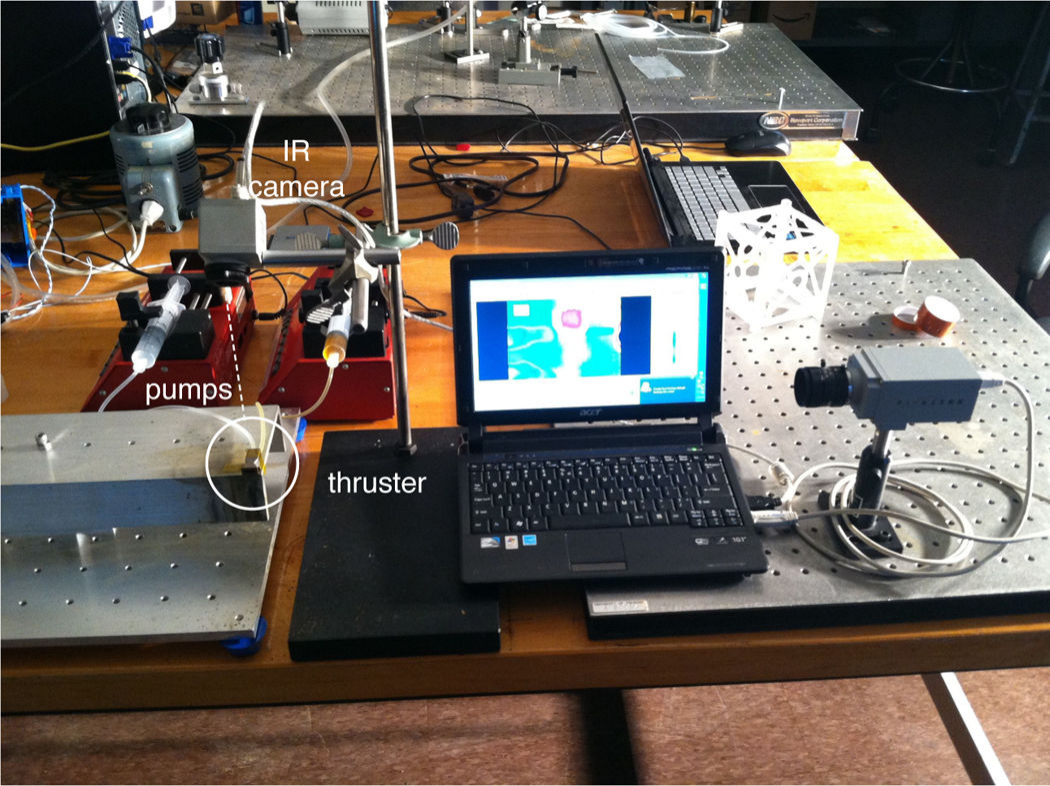
Photograph of thermal imaging set-up including thermal imaging camera, PixeLink camera, syringe pumps, and DMLS thruster.

Thermal imaging was used to visualize the temperature distribution within the device along with the plume at the nozzle exit during operation. A photograph of the experimental set-up is shown in Figure 11. Images were captured during steady-state operation for the plume and the thruster and subsequently examined. During operation in the supersonic regime, the temperature of the working fluid after exiting the nozzle is significantly lower than the temperature upstream of the nozzle and within the reaction chamber. In particular, the thermal image of the plume was used to determine the shape, direction, and temperature characteristics of the flow after exiting the nozzle. From this information, the ratio of catalyst solvent to hydrogen peroxide as well as the total flow rate of gases through the nozzle were examined to determine how these parameters affect the operation of the propulsion system.

Thermal imaging of the system was performed using a thermoIMAGER TIM T900 manufactured by Micro-Epsilon (Raleigh, NC USA). Thermal images were collected and analyzed using Micro-Epsilon’s thermal imaging software, TIM Connect. Along with thermal images, standard video was recorded using a PixeLink PL-B774U Color Camera. The thermal imaging camera was supported above the thruster, while the PixeLink camera was mounted to capture the side view of the thruster. Although the thermal camera was not calibrated to measure precisely the absolute temperatures of the materials, the thermal images nonetheless provide invaluable insight in the flow dynamics and operation of the system.

### 5.3. Exit Plane Temperature Measurement

The measurement of the temperature of the working fluid at the exit plane of the nozzle can be used to verify that the flow within the nozzle is in the supersonic regime. Supersonic flow through the nozzle is achieved if the pressure drop over the nozzle is high enough to choke the flow at the nozzle throat. As the nozzle diverges, a supersonic flow will accelerate as the flow area increases. The flow through a converging–diverging nozzle can be described using the well-known quasi-one-dimensional isentropic flow relation.

(3) $${\left(\frac{A}{A^{*}}\right)}^{2}=\frac{1}{M^{2}}{\left[\frac{2}{\gamma +1}\left(1+\frac{\gamma -1}{2}M^{2}\right)\right]}^{\frac{\gamma +1}{\gamma -1}}$$

Equation (3) relates the Mach number, *M*, the ratio of the flow area to the area of the throat, *A*/*A∗*, and the specific heat ratio of the gases flowing through the nozzle, *γ*. From this relationship, the geometry of the nozzle can be used to determine a theoretical Mach number at the nozzle exit plane *M*_*e*_ Using the temperature relation for isentropic supersonic flow,

(4) $$\frac{T_{e}}{T_{0}}={\left(1+\frac{\gamma -1}{2}{M_{e}}^{2}\right)}^{-1}$$

at this exit Mach number, the ratio of the stagnation temperature *T*_0_ to the exit temperature *T*_*e*_ can be calculated.

The adiabatic flame temperature of the reactants for each trial is the approximate stagnation temperature in the system. From this, it is possible to determine a theoretical working fluid temperature at the exit plane of the nozzle. These calculations result in the lowest achievable fluid temperature at the exit plane during experimentation due to the isentropic assumptions made in the calculation of the fluid exit temperature. Further, these temperature calculations allow the experimental exit plane temperature to be compared to the theoretical (isentropic) temperature. For these experiments, the catalyst solution used was 15 weight % aqueous ferric chloride. The ferric chloride itself was a laboratory grade anhydrous powder. The catalyst solution was prepared less than an hour prior to testing to eliminate any possible catalyst degradation due to oxygen exposure. Hydrogen peroxide used for the experiment was HTP manufactured by the FMC Corporation (Philadelphia, PA USA); the concentration was measured as 87.6 % using a Brix refractometer. With this combination of aqueous catalyst and HTP, the maximum adiabatic flame temperature expected during steady-state operation of the thruster was 685 K.

**Figure 12.**
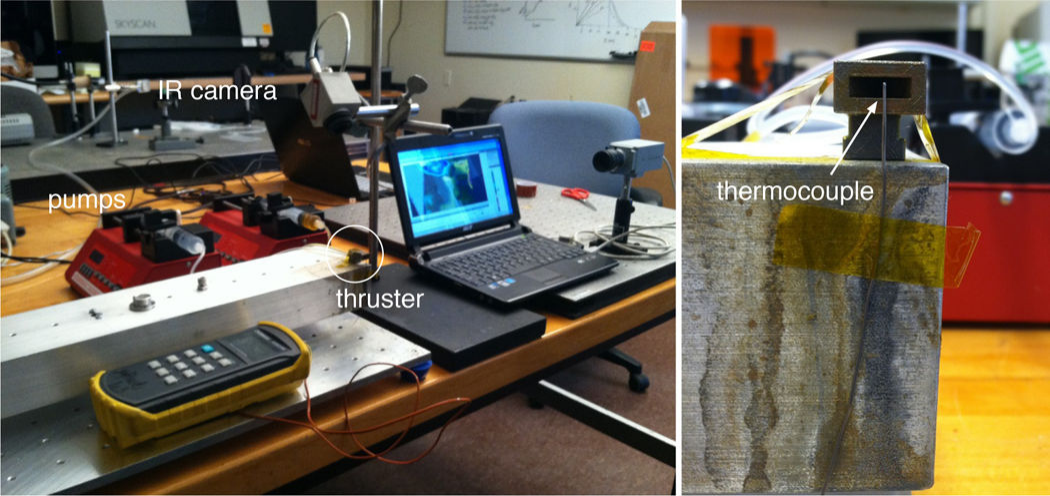
(**Right**) Photograph of DMLS stainless steel nozzle used for plume temperature measurement. A type K thermocouple was placed directly in the center of the nozzle exit for temperature measurements. (**Left**) Photograph of entire experimental set-up including thermal imaging camera, video camera, and digital thermometer.

The exit plane temperature measurement was performed using an Omega digital thermometer, model HH501AK, and a type K thermocouple. The thermocouple was placed directly at exit plane of the nozzle so that the end of the thermocouple was in the center of nozzle exit. The thermal imaging system was also used to capture thermal images of the plume during the tests. Along with thermal images, standard video was recorded using a PixeLink PL-B774U color camera. The experimental set-up for this experiment was similar to the arrangement used for the thermal imaging of the plume experiment and is shown in Figure 12.

### 5.4. Thrust Stand Measurement

Measurements of thruster performance were taken on a custom-built torsional thrust stand, with a sensitivity of 1.0 mN and a sampling rate of 20,000 samples/s. The position of the torsion arm is measured by a Digital Voltage Reluctance Transmitter (DVRT, Lord MicroStrain, Williston, VT USA), and captured using a National Instruments Model USB-6001 Digital Acquisition System (National Instruments Corporation, Austin, TX USA) . The positional data were converted real-time into raw thrust data and recorded for post-processing. The flow rate of the hydrogen peroxide and catalyst solutions entering the prototype thrusters was controlled via syringe pump. The prototype thruster was attached to the thrust stand and data collection began once the hydrogen peroxide and catalyst solution entered the mixing chamber.

## 6. Experimental Results

### 6.1. Schlieren and Thermal Imaging

**Figure 13.**
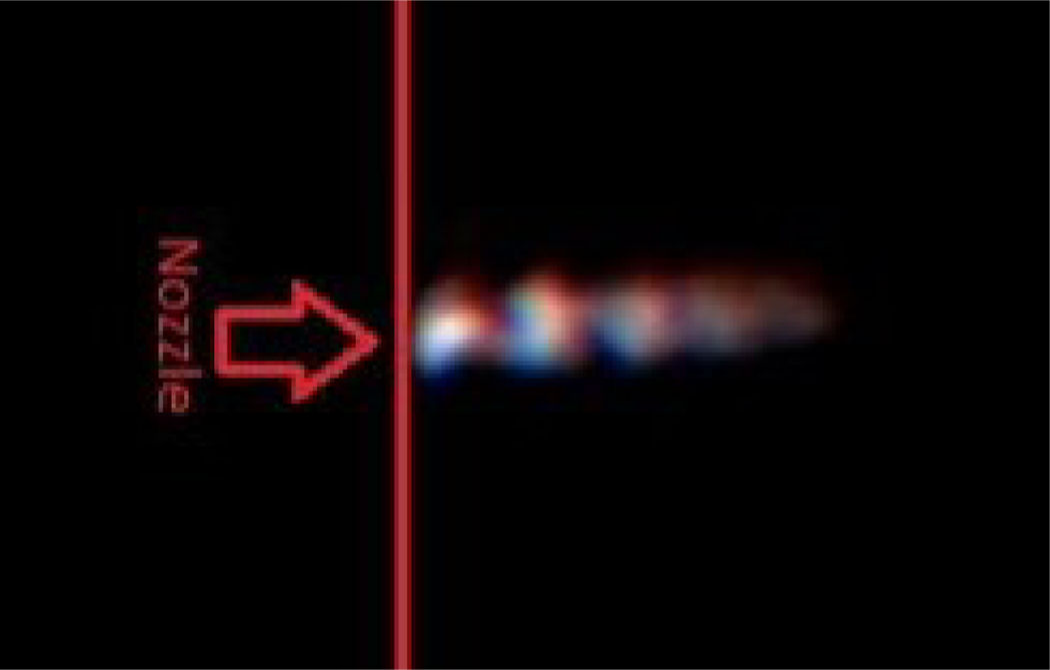
Schlieren photograph of ambient temperature air passing through the 30 degree half-angle nozzle vented directly to the laboratory, with an upstream stagnation pressure of approximately 55 psig. An overexpanded diamond shock pattern is visible which is evidence of supersonic flow in the diverging section of the nozzle.

An image obtained from Schlieren imaging appears in Figure 13 and shows an over-expanded diamond shock pattern in the flow exiting the nozzle; the over-expansion is a consequence of the ambient back-pressure in the experiments. The presence of the standing shock structure is direct evidence of supersonic flow at the nozzle exit.

The results obtained from the Schlieren imaging confirm that the nozzle operates properly with stagnation pressures as low as 55 psig. Due to the rapid decomposition and combustion reaction during operation, it is expected that the chemical propulsion system will have adequate pressure upstream of the nozzle to achieve supersonic flow in the diverging section of the nozzle when operating with the vacuum back-pressure conditions in space.

**Figure 14.**
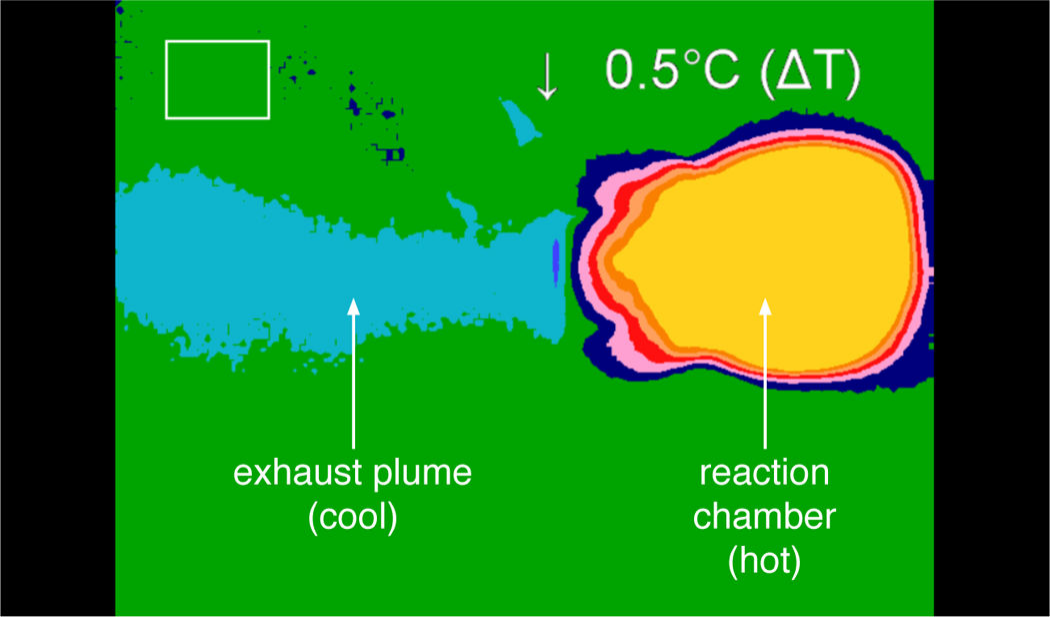
A thermal image of steady-state operation of the thruster using 15% aqueous ferric chloride as the catalyst solution and 87.6% HTP. The large temperature difference between the hot thruster reaction chamber (yellow) and cool exit plume (blue) is suggestive of supersonic operation.

**Figure 15.**
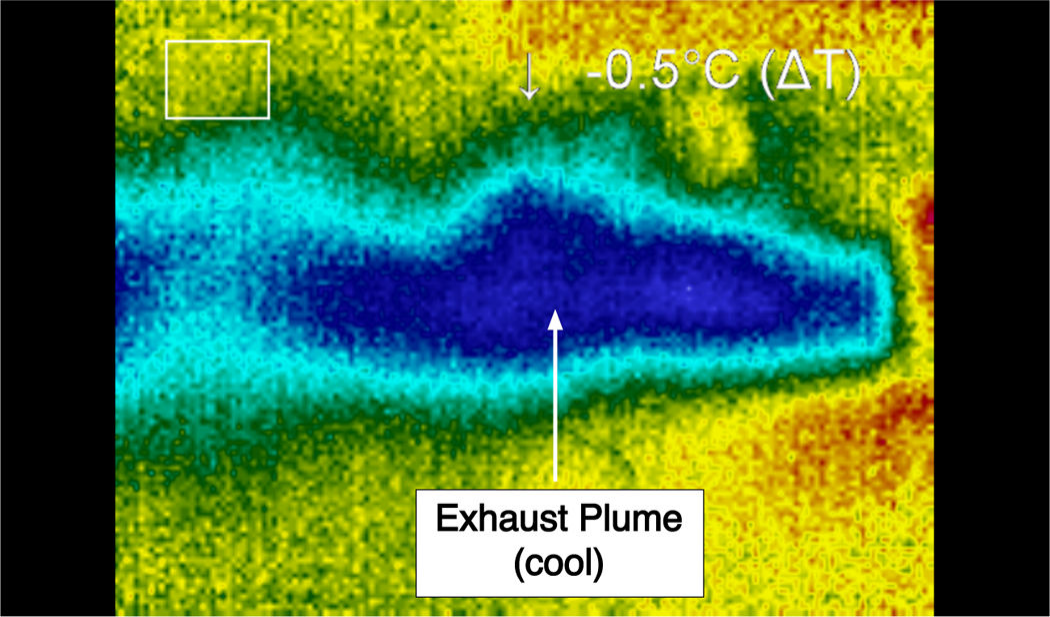
A stable plume created by the thruster at low gas flow rates. The blue region indicates the cooler temperature of the expanded flow within the plume.

Shown in Figure 14 is a thermal image that shows the temperature differential between the plume and the reaction chamber during thruster operation. In this image, the plume is blue, the surrounding laboratory atmosphere is green, and the reaction chamber is yellow. During steady-state operation, the temperature of the thruster chamber was essentially that of the stagnation temperature of the fluid upstream of the nozzle throat. This significant decrease in temperature along the flow through the device is consistent with supersonic operating conditions. Figure 15 provides supplementary thermal information of spatial higher resolution within the plume region. At these low gas flow rates, the thermal image of the plume was stable during steady-state operation.

**Figure 16.**
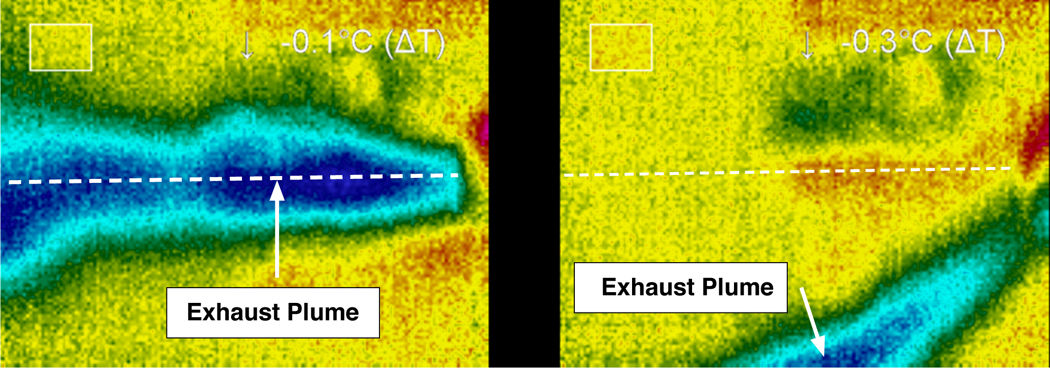
A flow instability at high flow rates through the thruster system. These instabilities are likely caused by incomplete mixing and reaction upstream of the nozzle.

At high flow rates, the plume developed instabilities which caused the direction of the plume to oscillate between the walls and the center of the nozzle (Figure 16). When the flow direction changed, no noticeable changes in plume temperature or flow patterns were observed suggesting that the nozzle remained within the supersonic flow regime regardless of the flow instability. It is posited that the instability in the flow direction was caused by incomplete mixing and/or chemical decomposition in the thruster’s reaction chamber; this in turn would result in suspended liquid droplets and a multiphase flow through the nozzle at high operational flow rates. The basis for this speculation is rooted in the numerical simulations by Greenfield et al. [23] who demonstrated that multiphase flow within supersonic micronozzles at sufficiently large Stokes numbers can produce plume deflection.

### 6.2. Exit Plane Temperature Measurements

**Figure 17.**
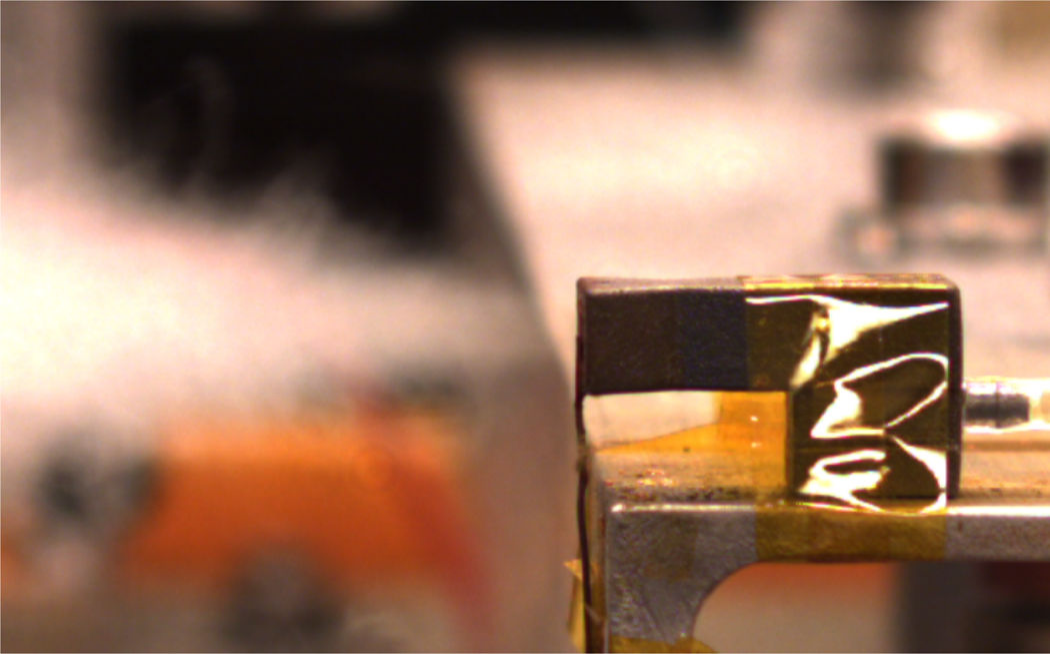
A photograph of the thruster system operating during the plume temperature measurement experiment. Condensation surrounding the plume is visible.

Figure 17 shows a photograph of the thruster during the plume temperature measurement experiment. In this photograph, the thermocouple placement in the center of the nozzle exit can be seen. Condensation of steam produced by the reaction is visible around the nozzle exit plume.

**Figure 18.**
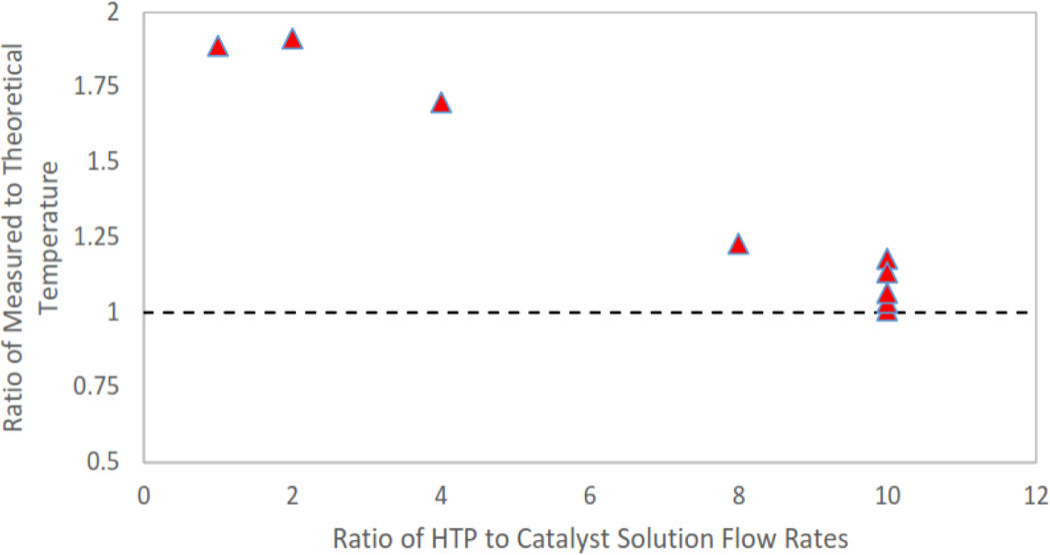
The ratio of measured to theoretical temperature vs. the ratio of volumetric flow rate of HTP to catalyst solution. High ratios of HTP to catalyst solution show agreement with quasi-one-dimensional isentropic supersonic flow in the nozzle. This strongly suggests the flow through the nozzle is choked at the throat at high HTP to catalyst solution ratios.

Using the quasi-1D relations in Equations (3) and (4), the measured temperatures were normalized by the idealized supersonic isentropic fluid temperature at the exit plane. The resulting ratio is plotted against the ratio of the flow rate of HTP to catalyst solution for each run in Figure 18. This plot shows that the measured temperatures of the working fluid at the nozzle exit agree within 18% of the calculated theoretical isentropic temperature when a volumetric ratio of 10 units HTP to 1 unit of catalyst was used. The closest measured temperature at this ratio agreed within 3.5% of the theoretical value. Along with the strong agreement with theoretical values at high volumetric ratios of HTP to catalyst solution, it is noteworthy that none of the recorded temperatures were below the minimum isentropic values.

The correlation observed between the experimental values and those calculated from the isentropic nozzle theory suggests that the nozzle design and mixing scheme were performing as expected during steady-state operation. These data also suggest that, for the nozzle size scale being tested, the Reynolds numbers were sufficiently large that the viscous boundary layers within the nozzle expander do not significantly impact the supersonic flow. This is in agreement with the numerical predictions by Louisos and Hitt for supersonic micronozzles [22] at the Reynolds numbers for these flows; however, future reduction in device size with much lower Reynolds numbers will likely reveal the presence of viscous impacts.

**Figure 19.**
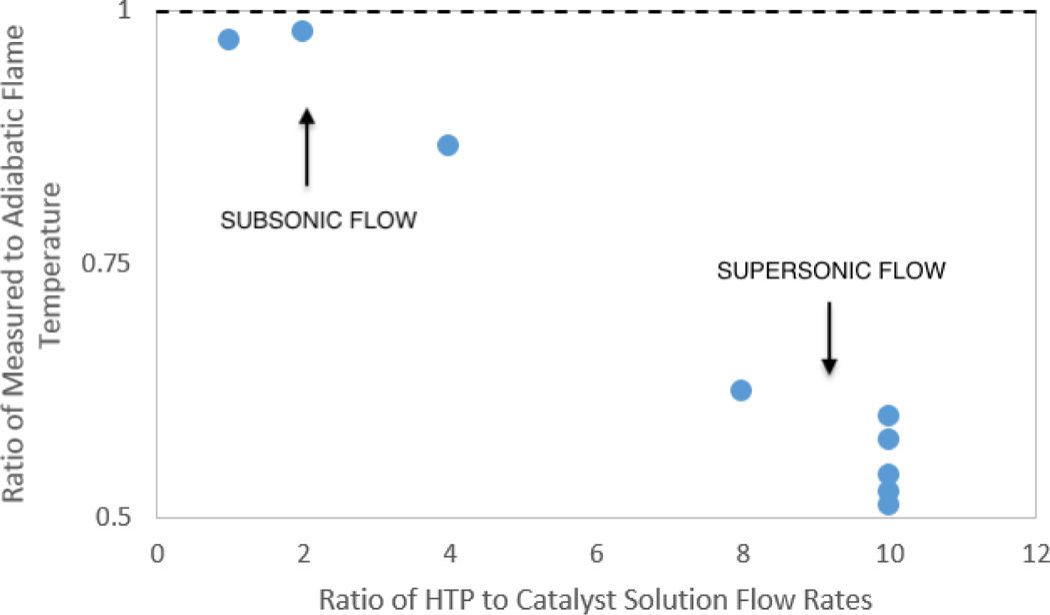
The ratio of measured to adiabatic flame temperature vs. the ratio of volumetric flow rate of HTP to catalyst solution. These data strongly suggest that, at low ratios of HTP to catalyst solution, flow is not choked at the nozzle throat and remains in the subsonic regime throughout the nozzle.

In an effort to further evaluate whether the flow within the thruster became supersonic, the ratio of the measured plume temperature to the adiabatic flame temperature was also computed; these data appear in Figure 19. The data show that low ratios of HTP to catalyst solution (1:1, 2:1, and 4:1) resulted in measured nozzle exit plane temperatures only slightly below adiabatic flame temperature. This indicates a flow that remains subsonic. The interpretation is that flows with low ratios of HTP to catalyst solution were not able to generate enough energy to create the necessary stagnation pressure to achieve choked flow at the throat. In contrast, cases of high ratios of HTP to catalyst solution (8:1 and 10:1) demonstrated a clear drop in exit temperature, as would be expected in supersonic flow.

### 6.3. Thrust Stand Measurements

**Figure 20.**
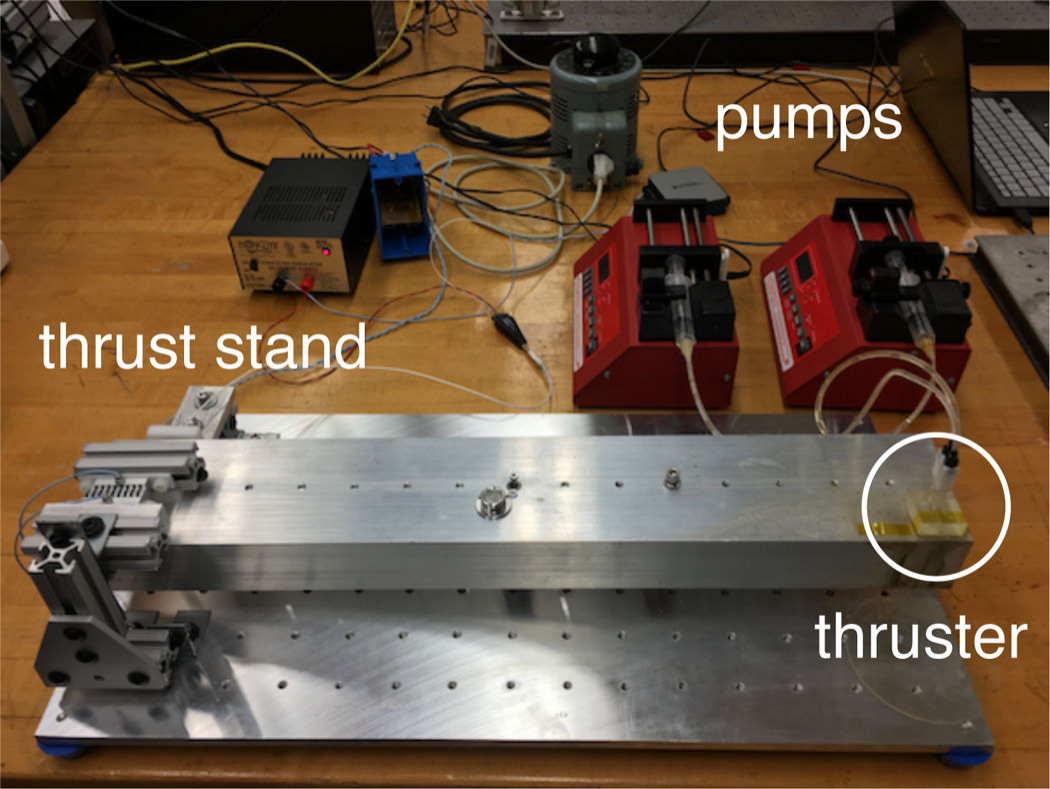
Photograph of the thrust stand apparatus.

**Figure 21.**
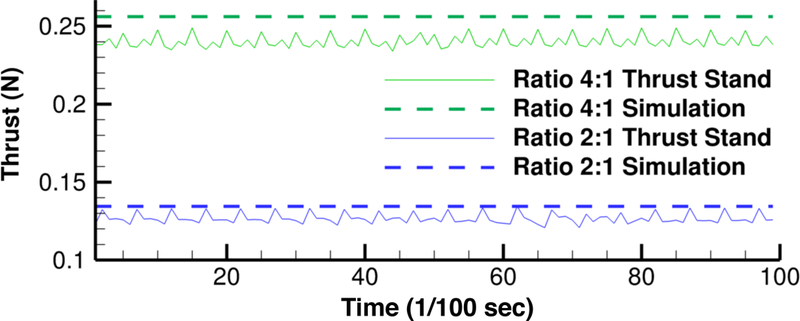
Thrust as a function of time for two different oxidizer:fuel ratios.

Thrust measurements of the device operating in steady-state were collected using the thrust stand apparatus shown in Figure 20. Data collected from the thrust stand measurements appear in Figure 21 for two different fuel mixture ratios. Predicted levels of thrust from nozzle theory are also included for comparison. The lower ratio (2:1) represents the system operating in pseduo-monopropellant mode, while the higher ratio (4:1) is operating in a range between monopropellant and bipropellant mode. For both ratios, the measured thrust is similar to the calculated thrust level, demonstrating that the system performance is in line with the expectations.

## 7. Conclusions

The thruster system outlined in this paper is a novel solution to providing attitude control and primary propulsion for the CubeSat platform at low cost. By leveraging microscale effects, the total mass and volume of the proposed propulsion system is less than similar, commercially available, CubeSat propulsion systems. The chemical propulsion system described in this paper has the ability to operate in both “psuedo-monopropellant” and “bipropellant” modes, which increases its flexibility for mission planning. The psuedo-monopropellant mode operation offers lower impulse bits while the bipropellant mode offers higher thrust. The thrust generated is controlled by adjusting the ratios of catalyst solution to hydrogen peroxide fed to the thrusters, the smallest ratio of catalyst solution to hydrogen peroxide that allows for decomposition of the hydrogen peroxide is used for the psuedo-monopropellant mode and a stoichiometric ratio of catalyst solution to hydrogen peroxide is used for the bipropellant mode. The bipropellant mode further takes advantage of hydrogen peroxide as an oxidizer that oxidizes the catalyst solvent resulting in a combustion reaction.

The prototype in this study was designed for operation and testing under ambient conditions. Future design modifications necessary for space operations involve only nozzle performance optimization, as the propellant reaction process is unaffected by ambient conditions. Specifically, the nozzle expansion ratio and chamber pressure will need to be adjusted to provide the target thrust level under vacuum exit conditions.

## Figures and Tables

**Table 1.** Operating parameters for high and low impulse modes. *I*_*sp*_ calculations are performed assuming a perfect converging–diverging nozzle, thrust is calculated using flow rates that will produce 50 mN of thrust during low impulse operation, impulse bit is calculated using a 10 ms valve actuation time, and Delta-V is calculated for a 3U CubeSat with 1U dedicated to fuel and oxidizer.

	I_SP_ (S)	Thrust (mN)	Impulse Bit (mN*s)	Delta-V (m/s)
High Impulse Mode*	291	112	11.2	986
Low Impulse Mode	161	50	5	574
Percent Difference	57.5	76.5	76.5	52.8

* 2-Propanol as catalyst solvent

**Table 2.** A table of observed solubility and reaction characteristics of experimental solutions. Solutions in white cells show high levels of solubility and reaction performance and are promising mixtures for further experimentation.

	Fe(lll)Cl_3_			Fe(ll)Cl_2_		
	5%	15%	25%	5%	15%	25%
Hexane	1	1	1	1	1	1
1-Hexanol	H, NC	H, NC	H, NC	NH, OSL	NH, OSL	NH, OSL
Methanol	H, NC	H, NC	H, NC	NH, NC	H, NC	H, UC
Ethanol	H, NC	H, NC	H, NC	H, NC	H, NC	NH, OSL
2-Propanol	H, NC	H, NC	NH, OSL	NH, OSL	NH, OSL	NH, OSL
Water	Frozen, NC	H, NC	H, NC	Frozen, NC	H, NC	NH, OSL

H=homogeneous

NH= not homogeneous

NC=noncolloidal, fully dissolved

UC = unstable colloid

OSL = over solubility limit

I = insoluble

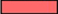
Long delay, slow reaction, or Insoluble

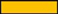
short delay followed by fast reaction or unstable colloid

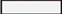
No delay, Fast reaction, Noncolloidal

**Table 3.** Summary of Key Propulsion System Parameters.

System Parameter	ADCS	Primary Divert (Bipropellant)
Nominal Thrust (N)	0.05	1.25
Length (mm)	26	27.46
Width (mm)	12	11.62
Nozzle Half Angle (Degrees)	30	15.53
Area Ratio	6	21
Chamber Pressure (kPa)	100	250
Chamber Temperature (K)	800	2200
Mass Flow Rate (g/s)	0.54	0.81
Reynolds Number	6500	10,000
